# TRPM7 kinase mediates hypomagnesemia-induced seizure-related death

**DOI:** 10.1038/s41598-023-34789-2

**Published:** 2023-05-15

**Authors:** Man Liu, Hong Liu, Feng Feng, Esther Krook-Magnuson, Samuel C. Dudley

**Affiliations:** 1 Cardiovascular Division, Department of Medicine, The Lillehei Heart Institute, University of Minnesota at Twin Cities, 2231 6th Street SE, CCRB 4-141, Minneapolis, MN 55455 USA; 2 Department of Neuroscience, University of Minnesota at Twin Cities, Minneapolis, MN USA

**Keywords:** Neuroscience, Physiology, Medical research, Molecular medicine, Neurology

## Abstract

Hypomagnesemia (HypoMg) can cause seizures and death, but the mechanism is unknown. Transient receptor potential cation channel subfamily M 7 (TRPM7) is a Mg transporter with both channel and kinase function. In this study, we focused on the kinase role of TRPM7 in HypoMg-induced seizures and death. Wild type C57BL/6J mice and transgenic mice with a global homozygous mutation in the TRPM7 kinase domain (TRPM7^K1646R^, with no kinase function) were fed with control diet or a HypoMg diet. After 6 weeks of HypoMg diet, mice had significantly decreased serum Mg, elevated brain TRPM7, and a significant rate of death, with females being most susceptible. Deaths were immediately preceded by seizure events. TRPM7^K1646R^ mice showed resistance to seizure-induced death. HypoMg-induced brain inflammation and oxidative stress were suppressed by TRPM7^K1646R^. Compared to their male counterparts, HypoMg female mice had higher levels of inflammation and oxidative stress in the hippocampus. We concluded that TRPM7 kinase function contributes seizure-induced deaths in HypoMg mice and that inhibiting the kinase reduced inflammation and oxidative stress.

## Introduction

Hypomagnesemia (HypoMg, serum Mg < 0.7 mmol/L) is associated with afebrile seizures and epilepsy, especially in patients with mutations of magnesium (Mg) transporters such as transient receptor potential cation channel subfamily M 7 (TRPM7), TRPM6, and cyclin and CBS domain divalent metal cation transport mediator 2 (CNNM2)^1–5^. The mechanism for these seizures is unknown, but a role for low Mg is clear: long-term Mg supplementation shows significant protection for these patients against seizures, cognitive development, and motor development^1–4^. While low Mg is used to acutely model seizures ex vivo^6–10^, mechanisms behind low Mg-induced seizures in vivo are likely multifaceted. Our recent study shows that HypoMg alone without mutations in Mg carriers is sufficient to cause seizures and death in mice, with higher susceptibility in female mice^11^.

One possible mechanism by which HypoMg induces seizures is through enhanced brain inflammation and oxidative stress^12–14^. For example, inhibition of the NOD-, LRR- and pyrin domain-containing protein 3 (NLRP3) inflammasome protects against chemical-induced seizures and epilepsy^15–17^. Inflammation-induced oxidative stress and cell apoptosis have been reported also to contribute to seizures and brain injury^10,14,18^. Mitochondrial oxidative stress is reported to lead to mitochondrial respiration defects and result in epilepsy in animal models^19,20^. Our previous study has shown that the antioxidant mitoTEMPO improved survival rates^11^, suggesting mechanisms that may relate to oxidative stress.

Overexpression of TRPM7, a Mg transporter with a kinase function, has been shown to induce inflammation with NLRP3 activation^21^, elevated oxidative stress^22,23^, and neuron cell apoptosis^23^, all of which could contribute to neuronal damage, seizure activity and death. Ryazanova et al.^24^ has showed that Mg deficiency results in death. Transgenic mice with a global homozygous mutation K1646R in the TRPM7 kinase domain (TRPM7^K1646R^) had no kinase function, were resistance to HypoMg-induced death, and lived 3-times longer than wild type (WT) mice^24^, suggesting the importance of TRPM7 kinase function in HypoMg-induced changes. Here, we investigated the mode of death and the mechanisms by which TRPM7 kinase activity may contribute to the epilepsy phenotype in HypoMg. We examined TRPM7 expression levels, phosphorylation of TRPM7 kinase targets, brain inflammation, and oxidative stress in WT and kinase-inactive TRPM7^K1646R^ mice on a low-Mg diet.

## Results

### Seizure-induced deaths associated with HypoMg were prevented by TRPM7 kinase inhibition

**Figure 1:**
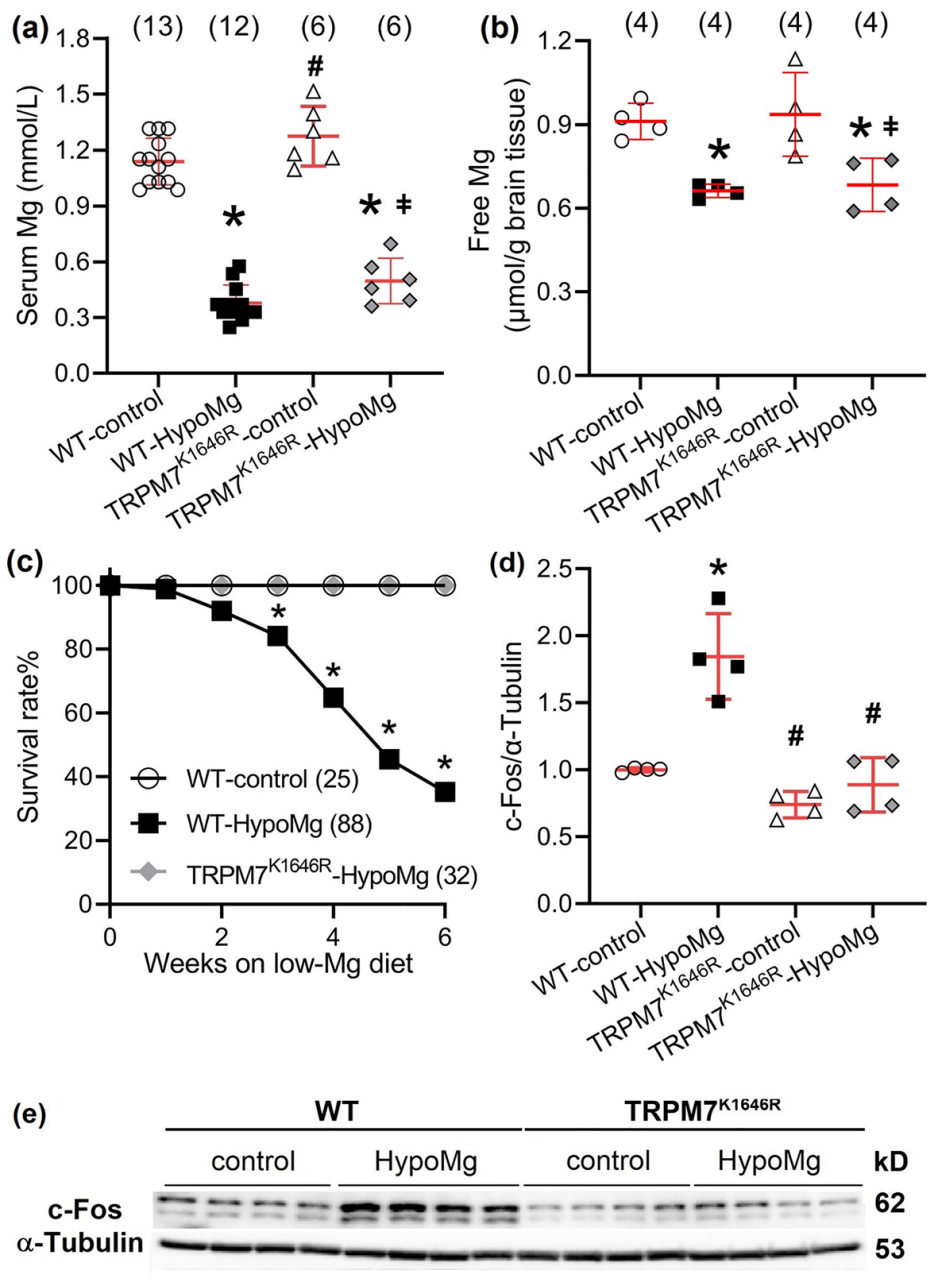
The low-Mg diet induced HypoMg, hippocampal hyperactivity, and death in mice. (**a**) HypoMg was induced by a low-Mg diet (15–30 mg/kg Mg, 6 weeks) in both WT and TRPM7^K1646R^ mice. Bars in the figure are mean ± SD. Two-way ANOVA multiple comparison test was used. *P < 0.001 vs. WT-control; ^#^P < 0.001 vs. WT-HypoMg; ^ǂ^P < 0.001 vs. TRPM7^K1646R^-control. The numbers of mice used for each group are shown in parentheses. (**b**) HypoMg induced decreased free Mg levels in WT- and TRPM7^K1646R^-HypoMg mouse brain tissue. Two-way ANOVA multiple comparison test was used. *P < 0.05 vs. WT-control; ^ǂ^P < 0.05 vs. TRPM7^K1646R^-control. The numbers of mice used for each group are shown in parentheses. (**c**) HypoMg-induced death was prevented by the mutation TRPM7^K1646R^ in mice. The log-rank (Mantel–Cox) test was applied for comparison between the WT-HypoMg and the WT-control or the TRPM7^K1646R^-HypoMg group. The numbers of mice used for each group are shown in parentheses. *P < 0.001 vs. WT-control or TRPM7^K1646R^-HypoMg (Fisher’s exact test: P = 0.02 at week 3, P < 0.001 at week 4–6). (**d,e**) c-Fos was significantly increased in the hippocampus of WT-HypoMg mice and normalized in that of TRPM7^K1646R^-HypoMg mice. Four mice were tested in each group. Western blot band intensities were normalized by the loading control α–tubulin and WT-control. Uncropped blots are in Supplementary Files. Bars in the figure are mean ± SD. Two-way ANOVA multiple comparison test was used for statistical analysis. *P < 0.001 vs. WT-control; ^#^P < 0.001 vs. WT-HypoMg. Half male and half female mice were tested in each group, except for the WT-HypoMg mice that were all male mice. *WT* wild type, *Control* mice with normal diet, *HypoMg* mice with the low diet, *TRPM7* transient receptor potential cation channel subfamily M 7, *TRPM7*^*K1646R*^ TRPM7 with a mutant K1646R.

As we have reported previously^11^, a 6-week low-Mg diet (15–30 mg/kg Mg) caused HypoMg with significantly decreased serum Mg (0.38 ± 0.03 vs. 1.14 ± 0.03 mmol/L for WT-control with normal diet of 2 g/kg Mg, P < 0.001, Fig. 1a) and death (more than 64%) in WT mice. Brain tissue free Mg levels were also decreased in the WT-HypoMg and TRPM7^K1646R^-HypoMg groups (Fig. 1b, Table 1) when compared with WT-control or TRPM7^K1646R^-control mice. Based on observation and electrocardiogram (ECG) telemetry recording, mice died immediately following seizure events (Supplementary Fig. S1 for representative telemetry trace and representative video clips of Supplementary Video 1 documenting seizures as the cause of death), a known complication of HypoMg^25^. Despite still suffering significant HypoMg (0.50 ± 0.05 mmol/L, P < 0.001 vs. WT-control, P = 0.31 vs. WT-HypoMg, Fig. 1a), all TRPM7^K1646R^ mice without TRPM7 kinase activity survived 6 weeks of the low-Mg diet (Fig. 1c). The levels of c-Fos, a biomarker for increased neuronal activity^26,27^, were significantly increased in the hippocampus of WT-HypoMg mice (1.85 ± 0.16-fold of WT-control, P < 0.001) and normalized in TRPM7^K1646R^ mice (0.89 ± 0.10-fold of WT-control, P = 0.85; P < 0.001 vs. WT-HypoMg) as shown in Fig. 1d,e and Table 1. This suggests that neuronal hyperactivity was at least partially corrected in TRPM7^K1646R^ mice.

**Table 1:** Summarized data of serum Mg, survival rate, and protein expression levels obtained from the whole brain tissue, except for c-Fos that were obtained from hippocampus.

	WT-control	WT-HypoMg	TRPM7^K1646R^-control	TRPM7^K1646R^-HypoMg
Serum Mg (mmol/L)	1.14 ± 0.03 (13)^11^	0.38 ± 0.03* (12)^11^	1.27 ± 0.07^†^ (6)	0.50 ± 0.05*^,^^‡^ (6)
Brain Mg (µmol per gram brain tissue)	0.91 ± 0.06 (4)	0.66 ± 0.02* (4)	0.94 ± 0.15 (4)	0.68 ± 0.10*^,^^‡^ (4)
Survival rate (%)	100% (25)^11^	64.8%* (88)^11^	100% (25)^†^	100%^†^ (32)
c-Fos (hippocampus)	1.00 ± 0.01	1.85 ± 0.16*	0.74 ± 0.05^†^	0.89 ± 0.10﻿^﻿†^
Mg and Ca^2+^transporters				
TRPM7	1.00 ± 0.07	1.43 ± 0.05*	0.97 ± 0.03^†^	1.00 ± 0.04^†^
MCU	1.00 ± 0.05	1.32 ± 0.06*	0.81 ± 0.08^†^	0.83 ± 0.10^†^
Inflammation				
NLRP3	1.00 ± 0.08	1.43 ± 0.07*	1.20 ± 0.08	1.07 ± 0.09^†^
ASC	1.00 ± 0.12	1.50 ± 0.13*	1.63 ± 0.06*	1.08 ± 0.08^†^^,^^‡^
IL-1β	1.00 ± 0.11	2.04 ± 0.44*	0.50 ± 0.18*^,^^†^	0.29 ± 0.07*^,^^†^
Oxidative stress				
Nitro-Tyr	1.00 ± 0.08	2.17 ± 0.09*	0.66 ± 0.04^†^	0.84 ± 0.05^†^
Protein phosphorylation				
p-PKCα	1.00 ± 0.07	1.24 ± 0.06*	1.18 ± 0.01	1.00 ± 0.05^†^
p-PKCδ	1.00 ± 0.04	1.31 ± 0.07*	1.02 ± 0.05	0.70 ± 0.07^†^^,^^‡^
p-Pyk2 (T402)	1.00 ± 0.04	1.35 ± 0.09*	1.01 ± 0.05^†^	0.87 ± 0.12^†^
p-Pyk2 (T579/580)	1.00 ± 0.08	1.59 ± 0.11*	0.77 ± 0.02^†^	1.03 ± 0.12^†^
p-RyR2 (S2808)	1.00 ± 0.10	2.04 ± 0.10*	2.17 ± 0.22*	0.73 ± 0.03*^,^^†^^,^^‡^

Mice were given normal (2 g/kg Mg) or low-Mg (15–30 mg/kg Mg diet and ddH_2_O as drinking water) diet for 6 weeks starting at 10-weeks old. For serum Mg, brain tissue free Mg, and survival rate, the numbers of mice used for each group are shown in parentheses. For protein tests, four mice were tested for each group. Protein levels were normalized first by the loading controls (α-tubulin or GAPDH) and then by the WT-control group. Data are mean ± SD. Two-way ANOVA multiple comparison test was used for statistical analysis.

*WT* wild type, *Control* mice with normal diet, *HypoMg* mice with the low diet, *TRPM7* transient receptor potential cation channel subfamily M 7, *TRPM7*^*K1646R*^ TRPM7 with a mutant K1646R, *p-PKC* phosphorylated protein kinase C, *p-RyR2* phosphorylated ryanodine receptor 2, *MCU* mitochondrial calcium uniporter, *p-Pyk2* phosphorylated protein tyrosine kinase 2, *NLRP3* the NOD-, LRR- and pyrin domain-containing protein 3, *ASC* apoptosis-associated speck-like protein containing a caspase recruitment domain, *IL-1β* interleukin-1β.

*P < 0.05 vs. WT-control.

^†^P < 0.05 vs. WT-HypoMg.

^‡^P < 0.05 vs TRPM7^K1646R^-control.

As reported previously^11^, all female mice were deceased within 6 weeks of the low-Mg diet (40 of 40), while male mice had a survival rate of 64.6% (Supplementary Fig. S2). Therefore, data reported at 6 weeks of WT-HypoMg is from exclusively male mice. Data from 4 weeks of HypoMg in “Sex differences in HypoMg-induced death” reflects equal numbers of age-matched male and female mice.

### TRPM7 kinase modulated select Mg transporter elevations in response to HypoMg

**Figure 2:**
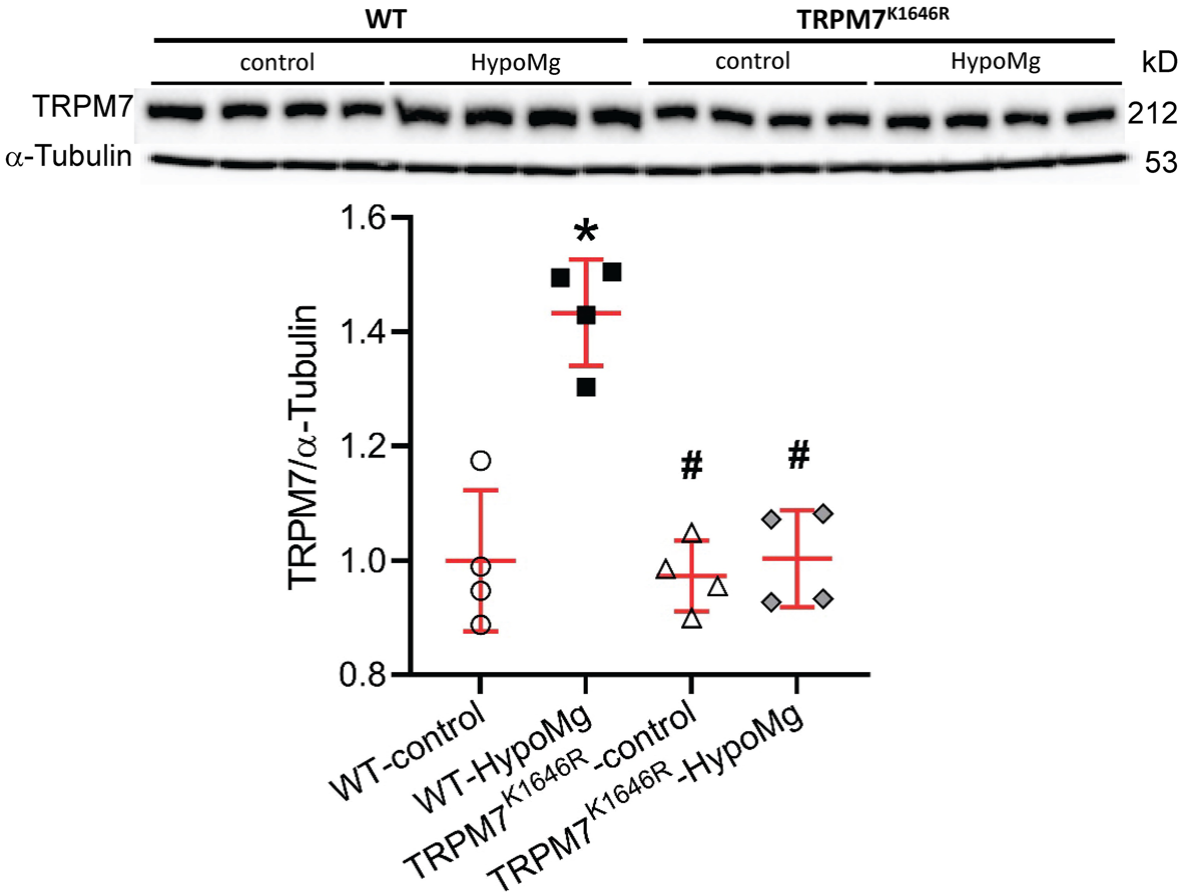
TRPM7 protein levels were increased in the WT-HypoMg mouse brain tissue and normalized in the TRPM7^K1646R^-HypoMg group. Western blot band intensities were normalized by the loading control α-tubulin and WT-control. Uncropped blots are in Supplementary Files. Four mice were tested in each group. Bars in the figure are mean ± SD. Two-way ANOVA multiple comparison test was used for statistical analysis. *P < 0.001 vs. WT-control; ^#^P < 0.001 vs. WT-HypoMg. Half male and half female mice were tested in each group, except for the WT-HypoMg mice that were all male mice. *WT* wild type, *Control* mice with normal diet, *HypoMg* mice with the low diet, *TRPM7* transient receptor potential cation channel subfamily M 7, *TRPM7*^*K1646R*^ TRPM7 with a mutant K1646R.

As shown in Fig. 2, the WT-HypoMg mouse brains had significantly increased expression of TRPM7 (1.43 ± 0.05-fold of WT-control, P < 0.001, Table 1). This was prevented by the mutant TRPM7^K1646R^ (1.00 ± 0.04-fold of WT-control, P < 0.001 vs. WT-HypoMg). We also measured Mg transporters CNNM2, mitochondrial RNA splicing 2 protein (MRS2), solute carrier family 41A1 (SLC41A1), and SLC41A3. CNNM2 and MRS2 were elevated in WT-HypoMg mouse brain tissue (CNNM2: 4.58 ± 0.51-fold of WT-control, P < 0.001; MRS2: 1.61 ± 0.12-fold of WT-control, P = 0.03) and normalized by TRPM7^K1646R^ (CNNM2: 0.84 ± 0.04-fold of WT-control, P = 0.97, P < 0.001 vs. WT-HypoMg; MRS2: 0.83 ± 0.17-fold of WT-control, P = 0.66, P = 0.004 vs. WT-HypoMg). The two SLC41 transporters were not affected by HypoMg (SLC41A1: 0.97 ± 0.33-fold of WT-control, P > 0.99; SLC41A3: 0.84 ± 0.23-fold of WT-control, P = 0.56).

### TRPM7 kinase regulated HypoMg-induced neuronal inflammation

**Figure 3:**
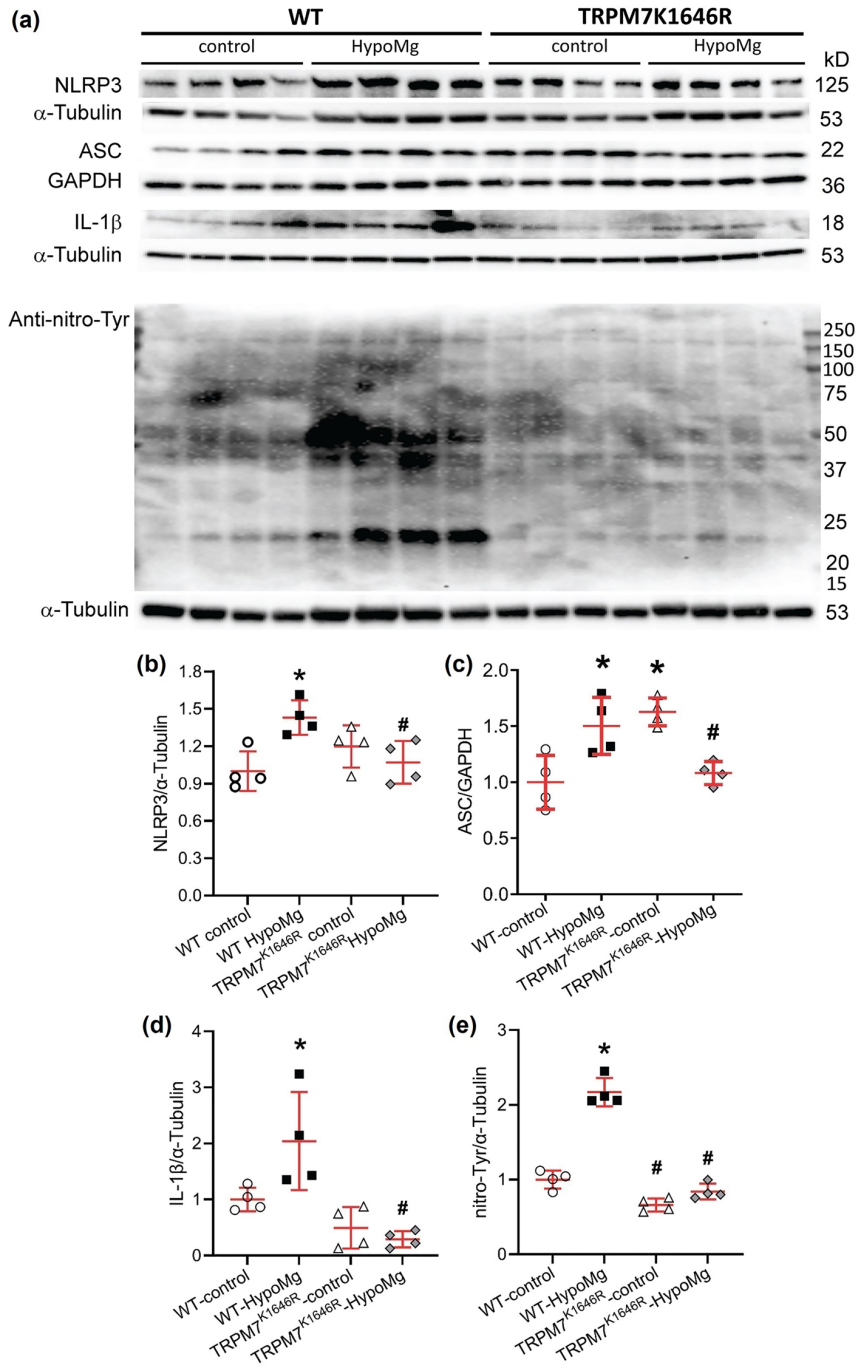
HypoMg induced (**a–d**) inflammation and (**a,e**) oxidative stress in WT mouse brain tissue, which were normalized by the mutation K1646R. (**a**) Western blot protein bands with molecular weights. Uncropped blots are in Supplementary Files. (**b–e**) Western blot bands intensities were normalized by loading controls (α-tubulin or GAPDH) and WT-controls. Four mice were tested in each group. Bars in the figures are mean ± SD. Two-way ANOVA multiple comparison test was used for statistical analysis. *P < 0.05 vs. WT-control; ^#^P < 0.05 vs. WT-HypoMg. Half male and half female mice were tested in each group, except for the WT-HypoMg mice that were all male mice. *WT* wild type, *Control* mice with normal diet, *HypoMg* mice with the low diet, *TRPM7* transient receptor potential cation channel subfamily M 7, *TRPM7*^*K1646R*^ TRPM7 with a mutant K1646R, *NLRP3* the NOD-, LRR- and pyrin domain-containing protein 3, *ASC* apoptosis-associated speck-like protein containing a caspase recruitment domain, *IL-1β* interleukin-1β.

NLRP3, apoptosis-associated speck-like protein containing a caspase recruitment domain (ASC), and pro-caspase-1 form the NLRP3 inflammasome, an important signaling complex central to innate immunity. The NLRP3 inflammasome contributes to activation of caspase-1 to produce mature interleukin-1β (IL-1β), a pro-inflammatory cytokine^28^. HypoMg induced brain inflammation, as shown in Fig. 3a–d and Table 1, with increased levels of NLRP3, ASC, and IL-1β. These elevations were normalized in TRPM7^K1646R^-HypoMg mouse brain tissue, despite ASC being elevated in TRPM7^K1646R^-control mice under normal diet.

### TRPM7 kinase regulated HypoMg-induced oxidative stress

HypoMg significantly increased protein oxidation with increased total protein nitro-tyrosine levels in WT-HypoMg brain tissues (2.17 ± 0.19-fold of WT-control, P < 0.001, Fig. 3a,e, Table 1). This oxidation was prevented by TRPM7 kinase inactivation (0.84 ± 0.11-fold of WT-control, P = 0.36, P < 0.001 vs. WT-HypoMg; Fig. 3e, Table 1).

### TRPM7 kinase modulated HypoMg-induced changes in kinase activation

**Figure 4:**
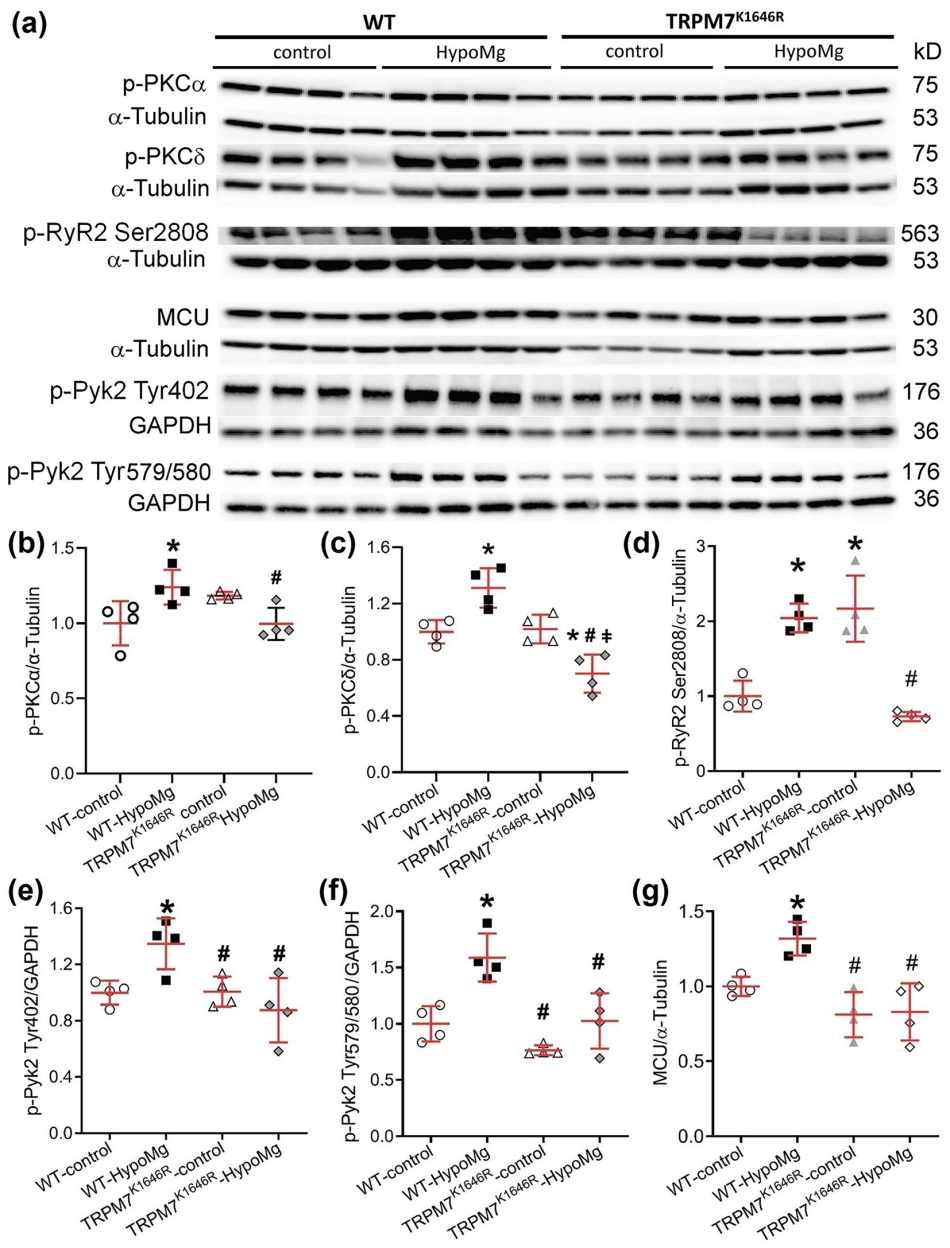
Phosphorylation levels of PKCα, PKCδ, RyR2 and Pyk2 and MCU protein levels were increased in the WT-HypoMg mouse brain tissue and reversed in the TRPM7^K1646R^ group. (**a**) Western blot protein bands with molecular weights. Uncropped blots are in Supplementary Files. (**b–g**) Western blot band intensities of were normalized by loading controls (α-tubulin or GAPDH) and WT-controls. Four mice were tested in each group. Bars in the figures are mean ± SD. Two-way ANOVA multiple comparison test was used for statistical analysis. *P < 0.05 vs. WT-control; ^#^P < 0.05 vs. WT-HypoMg; ^ǂ^P < 0.05 vs. TRPM7^K1646R^-control. Half male and half female mice were tested in each group, except for the WT-HypoMg mice that were all male mice. *WT* wild type, *Control* mice with normal diet, *HypoMg* mice with the low diet, *TRPM7* transient receptor potential cation channel subfamily M 7, *TRPM7*^*K1646R*^ TRPM7 with a mutant K1646R, *p-PKC* phosphorylated protein kinase C, *p-RyR2* phosphorylated ryanodine receptor 2, *MCU* mitochondrial calcium uniporter, *p-Pyk2* phosphorylated protein tyrosine kinase 2.

Increased phosphorylation of protein kinase C (p-PKC) has been reported to increase oxidative stress^29^ and cell apoptosis that can lead to seizure activities^13,30^. Phosphorylated protein tyrosine kinase 2 (p-Pyk2) phosphorylates and increases mitochondrial calcium uniporter (MCU) activity^31^, which has been linked to increased seizure activity^10^. Therefore, we tested whether HypoMg affected phosphorylation of PKCα/δ and Pyk2. We observed increased phosphorylation of PKCα, PKCδ (Fig. 4a–c, Table 1), and Pyk2 (at Tyr402 and Tyr579/S580, Fig. 4a,e,f, Table 1) in WT-HypoMg mouse brain tissue, which were all normalized in the TRPM7^K1646R^-HypoMg group. Furthermore, MCU protein levels were also significantly increased in WT-HypoMg mouse brain tissue and normalized in the TRPM7^K1646R^-HypoMg group (Fig. 4a,g, Table 1). The activation of kinases was not universal, however. The phosphorylation of c-Src (another reported kinase that phosphorylates MCU)^32^ was unaltered in WT-HypoMg mouse brain tissue (phospho-Src Ser17: 0.86 ± 0.15-fold of WT-control, P = 0.67).

### TRPM7 kinase contributed to HypoMg-induced RyR2 phosphorylation

Increased phosphorylation of ryanodine receptor 2 (p-RyR2) results in calcium (Ca^2+^) leak from the endoplasmic reticulum, which can contribute to seizure activity^33–36^. TRPM7 kinase inactivation prevented HypoMg-induced elevation of RyR2 phosphorylation at the Ser2808 (Fig. 4a,d, Table 1). The Ser2814 site, also associated with RyR2 leakiness, was unchanged by HypoMg or by TRPM7 kinase inhibition (WT-HypoMg: 1.12 ± 0.15-fold of WT-control, P = 0.94; TRPM7^K1646R^-control: 1.16 ± 0.28-fold of WT-control, P = 0.86).

### Sex differences in HypoMg-induced protein phosphorylation, inflammation, and oxidative stress

**Figure 5:**
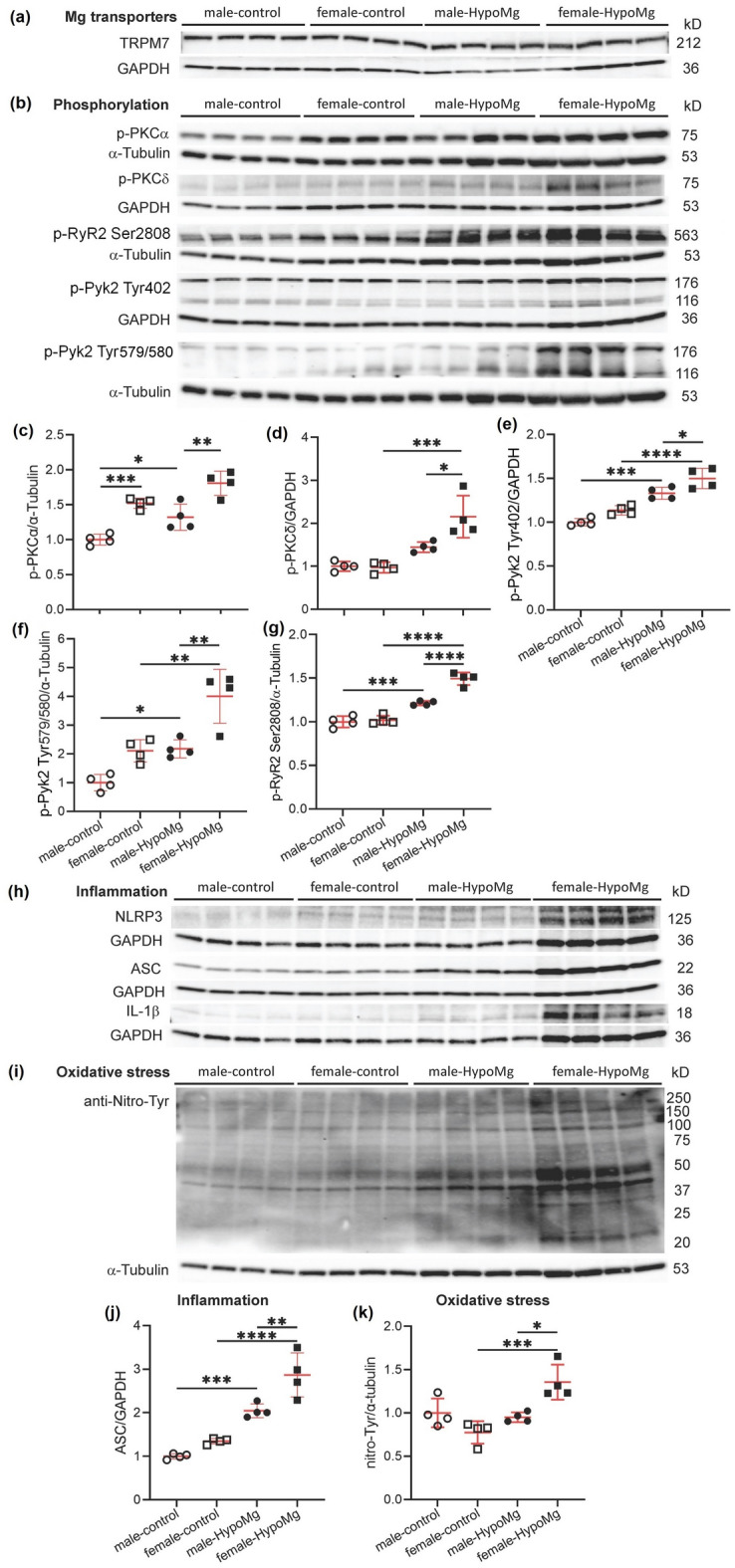
Sex differences observed in the seizure-caused death rates between males and females was mirrored in sex difference in (**b–g**) increased phosphorylation of PKCα/δ, RyR2 (S2808), and Pyk2 (T402 and S579/580), (**h,j**) increased inflammation (ASC), and (**i,k**) increased protein oxidation observed in the hippocampus. Mice were given normal (2 g/kg Mg) or low-Mg (15–30 mg/kg Mg diet and ddH_2_O as drinking water) diet for 4 weeks starting at 10-week old. (**a,b,h,i**) Western blot protein bands with molecular weights. Uncropped blots are in Supplementary Files. (**c–g,i–k**) Western blot bands intensities were normalized by loading controls (α-tubulin or GAPDH) and male-controls. Four mice were tested in each group. Bars in the figures are mean ± SD. Two-way ANOVA multiple comparison test was used for statistical analysis. *P < 0.05, **P < 0.01, ***P < 0.001. *Control* mice with normal diet, *HypoMg* mice with the low diet, *TRPM7* transient receptor potential cation channel subfamily M 7, *TRPM7*^*K1646R*^ TRPM7 with a mutant K1646R, *p-PKC* phosphorylated protein kinase C, *p-RyR2* phosphorylated ryanodine receptor 2, *MCU* mitochondrial calcium uniporter, *p-Pyk2* phosphorylated protein tyrosine kinase 2, *NLRP3* the NOD-, LRR- and pyrin domain-containing protein 3, *ASC* apoptosis-associated speck-like protein containing a caspase recruitment domain, *IL-1β* interleukin-1β.

Seizure-induced death in HypoMg mice is significantly higher in females than in males^11^. In our previous study^11^, all female mice (40 out of 40) died within 6-week treatment of the low-Mg diet, while 35.4% of males (17 out of 48) died (Supplementary Fig. S2). The Log-rank (Mentel-Cox) test gave a P < 0.001 when comparing WT-control and WT-HypoMg male, P < 0.001 when comparing WT-control and WT-HypoMg female, and P < 0.001 when comparing WT-HypoMg male and WT-HypoMg female. Consistent with our previous findings^11^, female and male mice had similar levels of serum and urine Mg after 4 weeks of the low-Mg diet (Table 2). To identify potential explanations for the sex difference, we compared hippocampus protein phosphorylation, inflammation, and oxidative stress after 4 weeks of the low-Mg diet. TRPM7 protein levels showed no sex difference (Fig. 5a, Table 2). The female HypoMg mouse hippocampus had higher levels of protein phosphorylation of PKCα, PKCδ, Pyk2 (Tyr402 and Tyr579/580), and RyR2 (S2808) (Fig. 5b–g), more inflammation as measured by ASC (Fig. 5h,j), and elevated protein oxidation (nitro-Tyr, Fig. 5i,k), compared with the HypoMg male hippocampus (Table 2). A trend of higher levels of NLRP3 and IL-1β were observed in female-HypoMg hippocampus when compared with the male-HypoMg group (Table 2).

**Table 2:** Sex differences in serum and urine Mg levels and protein expression of the hippocampus.

	Male-control	Female-control	Male-HypoMg	Female-HypoMg
Serum Mg (mmol/L)	1.07 ± 0.04 (11)	1.16 ± 0.04 (9)	0.36 ± 0.01* (7)	0.33 ± 0.04*^,^^†^ (9)
Urine Mg (mmol/L)	25.82 ± 1.81 (3)	37.95 ± 6.35 (3)	0.23 ± 0.07* (3)	0.16 ± 0.01*^,^^†^ (3)
Mg transporters				
TRPM7	1.00 ± 0.04	0.99 ± 0.07	1.35 ± 0.14	0.92 ± 0.22
Inflammation				
NLRP3	1.00 ± 0.09	1.04 ± 0.14	1.46 ± 0.12*	1.76 ± 0.12*^,^^†^
ASC	1.00 ± 0.03	1.35 ± 0.03	2.04 ± 0.08*	2.87 ± 0.25*^,^^†^^,^^‡^
IL-1β	1.00 ± 0.09	1.01 ± 0.09	2.32 ± 0.14*	2.72 ± 0.16*^,^^†^
Oxidative stress				
Nitro-Tyr	1.00 ± 0.08	0.78 ± 0.07	0.95 ± 0.03	1.36 ± 0.10*^,^^†^^,^^‡^
Protein phosphorylation				
p-PKCα	1.00 ± 0.04	1.52 ± 0.04*	1.32 ± 0.09*	1.81 ± 0.09*^,^^‡^
p-PKCδ	1.00 ± 0.06	0.97 ± 0.06	1.45 ± 0.06	2.16 ± 0.24*^,^^†^^,^^‡^
p-Pyk2 (T402)	1.00 ± 0.02	1.14 ± 0.03	1.33 ± 0.03*	1.50 ± 0.06*^,^^†^^,^^‡^
p-Pyk2 (T579/580)	1.00 ± 0.14	2.11 ± 0.20	2.18 ± 0.16*	4.01 ± 0.47*^,^^†^^,^^‡^
p-RyR2 (S2808)	1.00 ± 0.03	1.02 ± 0.03	1.21 ± 0.01*	1.49 ± 0.04*^,^^†^^,^^‡^

Mice were given normal (2 g/kg Mg) or low-Mg (15–30 mg/kg Mg diet and ddH_2_O as drinking water) diet for 4 weeks starting at 10-week old. For serum and urine Mg, the numbers of mice used for each group are shown in parentheses. For protein tests, four mice were tested in each group. Protein levels were normalized first by the loading controls (α-tubulin or GAPDH) and then by the male-control group. Data are mean ± SD. Two-way ANOVA multiple comparison test was used for statistical analysis.

*Control* mice with normal diet, *HypoMg* mice with the low diet, *TRPM7* transient receptor potential cation channel subfamily M 7, *NLRP3* the NOD-, LRR- and pyrin domain-containing protein 3, *ASC* apoptosis-associated speck-like protein containing a caspase recruitment domain, *IL-1β* interleukin-1β, *p-PKC* phosphorylated protein kinase C, *p-Pyk2* phosphorylated protein tyrosine kinase 2, *p-RyR2* phosphorylated ryanodine receptor 2.

*P < 0.05 vs. male-control.

^†^P < 0.05 female-HypoMg vs. female-control.

^‡^P < 0.05 female-HypoMg vs. male-HypoMg.

## Discussion

HypoMg has been long associated with increased seizure activities and epilepsy^1–4,37^. We have found that a low-Mg diet induces seizures and death, with female mice being more susceptible than males^11^. Neuronal tissues and cells treated with Mg-free media have been used to generate in vitro and ex vivo models of epileptiform activity^6–10^. Previous studies have been mainly focused on HypoMg-induced changes of synaptic receptors and their function, including glutamate receptors, like *N*-methyl-D-aspartate (NMDA) receptors, and gamma-aminobutyric acid (GABA) receptors^38,39^. However, in vivo additional mechanisms may significantly contribute to HypoMg-induced seizures and death.

Mutations in both TRPM6 and TRPM7 have been associated with HypoMg and seizures^2,4,5^. Moreover, Mg supplementation can prevent these seizures. In the above results, HypoMg induces seizures by increasing neural inflammation, and this provides an explanation for the clinical observation that Mg supplementation can reduce seizure risk in patients carrying mutations in these channels. In this study, we showed that the Mg transporter TRPM7 kinase function plays a fundamental role in HypoMg-induced seizure-related deaths, as TRPM7 kinase-inactive mice showed significantly improved survival rates (indeed, zero death was observed in TRPM7^K1646R^ mice over the course of this study). We found that TRPM7 kinase inhibition additionally reversed the increase in brain kinase activation, RyR2 activation, inflammation, and oxidative stress associated with HypoMg. Although other causes of seizures and death cannot be excluded, since each of these components is associated with seizures, these changes may contribute to why TRPM7 kinase inhibition prevented HypoMg-induced seizures and death. Therefore, TRPM7 kinase appears to be upstream of a cascade of changes that mediate hippocampal hyperexcitability and seizure-induced death in response to HypoMg. These results extended the findings of Ryazanova et al.^24^ that inhibition of the TRPM7 kinase function can inhibit death associated with low-Mg diet. In their study, mice lacking the TRPM7 kinase function eventually succumbed to HypoMg starting after ~ 60 days, suggesting that HypoMg may have deleterious effects independent of the TRPM7 kinase that develop over time. Since we did not follow mice for that length of time, it is impossible for us to say if the TRPM7^K1646R^-HypoMg group of mice would have eventually developed neural inflammation, seizures, and death or some other lethal process.

Mg deficiency has been linked to inflammation and oxidative stress for decades^12,40,41^. Inflammation and oxidative stress are associated with increased seizure activity^14,18,38^. For example, NLRP3 inflammasome is involved in epilepsy progression^15–17^. The NLRP3 inhibitors ametoflavone and CY-09 alleviate inflammation with decreased IL-1β and reduce seizure susceptibility in pentylenetetrazole-treated mice^16,17^. We observed inflammation and oxidative stress in HypoMg mouse brain tissue, which were eliminated by TRPM7^K1646R^. Similar to our study, a link between TRPM7 and inflammation was reported in an in vitro study on H9c2 cells, where TRPM7 overexpression induces NLRP3 activation^21^.

Consistent with our results, inhibition of TRPM7 has been shown to reduce ROS in cultured neuron cells^42^ and human hepatoma cells^43^. Oxidative stress is also reported to enhance TRPM7 activity^44,45^, which could form a positive feedback under HypoMg and exacerbate seizure activity. In our previous study, a mitochondrial targeted antioxidant mitoTEMPO improved survival in HypoMg^11^. This suggests that the mitochondrial ROS (mitoROS) could be a major source of oxidative stress induced by HypoMg, that mitoROS contributes to HypoMg-induced seizures and death, and that TRPM7 kinase can activate mitoROS overproduction. Dr. Patel’s group has reported in animal models of epilepsy that mitoROS in neuron cells cause mitochondrial respiration defects with significantly decreased mitochondrial maximal respiration and reserve capacity, leading to epilepsy^19,20^.

HypoMg-induced oxidative stress may be explained by increased mitochondrial Ca^2+^ flux secondary to Pyk2 activation and increased MCU activity. Inhibiting MCU has been shown to lower the rate of HypoMg-induced acquired epilepsy and decrease mitoROS production significantly in hippocampal neurons, while activating MCU has the opposite effects^10^. In our study, MCU activity was enhanced by HypoMg with elevated MCU protein expression and increased phosphorylation of Pyk2, a kinase which phosphorylates MCU and enhances its activity^31^. With inactivation of the TRPM7 kinase, both MCU protein expression and Pyk2 phosphorylation were suppressed, associated with normalized ROS levels (as measured by total protein oxidation) and normalized neuronal activity (as measured by hippocampal c-Fos levels).

Interestingly, HypoMg increased TRPM7 levels, and TRPM7 kinase seems to regulate TRPM7 level changes in response to HypoMg. The lack of increase in TRPM7 levels in response to HypoMg in kinase inhibited mice may be explained by the observations of Schmitz et al.^46^ who reported that the TRPM7^K1646R^ channel had significantly reduced sensitivity to Mg levels despite unchanged transmembrane conduction of Mg.

Increased TRPM7 at the mRNA and protein levels has been reported to play deleterious roles in brain injury and brain tumors^47,48^. Inhibitors of TRPM7 such as carvacrol and waixenicin A that block the channel function have shown protection against hypoxia/ischemic brain injury via promoting pro-survival signaling and suppressing pro-apoptotic signaling^23,47^. Suppression of hippocampal TRPM7 with shRNA has been shown to prevent neuron death in brain ischemia^23^. These lines of evidence suggest that elevated neuronal TRPM7 is deleterious.

In our study, we observed significantly increased TRPM7 protein levels under HypoMg. This increase was prevented by TRPM7 kinase inhibition. The lack of TRPM7 increase with inhibition of the kinase function may help explain the prevention of seizure-related deaths. At 4 weeks of HypoMg in kinase deficient mice, TRPM7 was not elevated, suggesting that the kinase function is likely more deleterious than the channel transport function initially. The kinase also appeared to be important in regulating CNNM2. We found that CNNM2 was increased by HypoMg in WT mice but not in inactive TRPM7 kinase mice. Bai et al.^49^ has shown that CNNMs bind to and can increase TRPM7 Mg transport. The changes in CNNM2 did not correlate with brain Mg content. Nevertheless, the results suggest that HypoMg affects CNNM levels by altering TRPM7 kinase activity.

TRPM7 kinase appeared to regulate a signaling cascade involved in seizure activity. In this study, we found significantly increased phosphorylation of PKCα and PKCδ under HypoMg, which were reversed by TRPM7^K1646R^. Increased PKC activity has been reported in status epilepticus and epileptogenesis: increased PKCδ activity and inflammation are observed in pilocarpine-induced epilepsy in rats^13^. PKC phosphorylation has been linked to increased release of glutamate (by blocking neuropeptide-Y-mediated inhibition of glutamate release)^30,50^, inflammation activation^13^, and increased oxidative stress^29^, all of which have been associated with increased seizure activities. TRPM7 kinase also affects Pyk2 phosphorylation, which could subsequently phosphorylate MCU^31^, enhance MCU activity and lead to increased seizure activities^10^. Since Pyk2 was phosphorylated at Tyr sites, it is likely that there is an intermediate kinase that is phosphorylated by TRPM7, since the TRPM7 is a Ser/Thr kinase.

Calcium signaling may also contribute to the observed epilepsy phenotype and death, as Ca^2+^ signaling plays important roles in seizures and epilepsy. Increased phosphorylation of RyR2 can lead to RyR2 Ca^2+^ leak^27,51^, and leaky RyR2 has been linked to seizures, epilepsy, and sudden death^33–36^. In our study, we observed increased phosphorylation of RyR2 in the HypoMg mouse brain tissues. TRPM7 kinase inhibition suppressed RyR2 phosphorylation in response to HypoMg. It is unclear whether TRPM7 phosphorylates RyR2 directly or through a signaling cascade. Given the importance of the TRPM7 kinase to the pathology of HypoMg, it will likely prove useful to determine the TRPM7 kinase targets and signaling cascade when designing strategies to mitigate the effects of HypoMg. All three RyR isoforms, RyR1, RyR2, and RyR3, are expressed in the brain, with RyR2 predominating^52^. We did not measure the phosphorylation of RyR1 or RyR3, and therefore, could not rule out any possible roles of RyR1 and RyR3 in HypoMg-induced seizure activity.

Sex differences in HypoMg-induced death were accompanied by higher levels of kinase activity, RyR2 phosphorylation, oxidative stress, and inflammation in female mice. It is possible that similar mechanisms contribute to HypoMg-induced seizures and death in male and female mice but female mice developed these changes earlier than male mice. Female mice seem primed to respond to HypoMg since female mice showed higher levels of p-PKCα, p-Pyk2, and ASC than male mice at baseline.

## Conclusion

**Figure 6:**
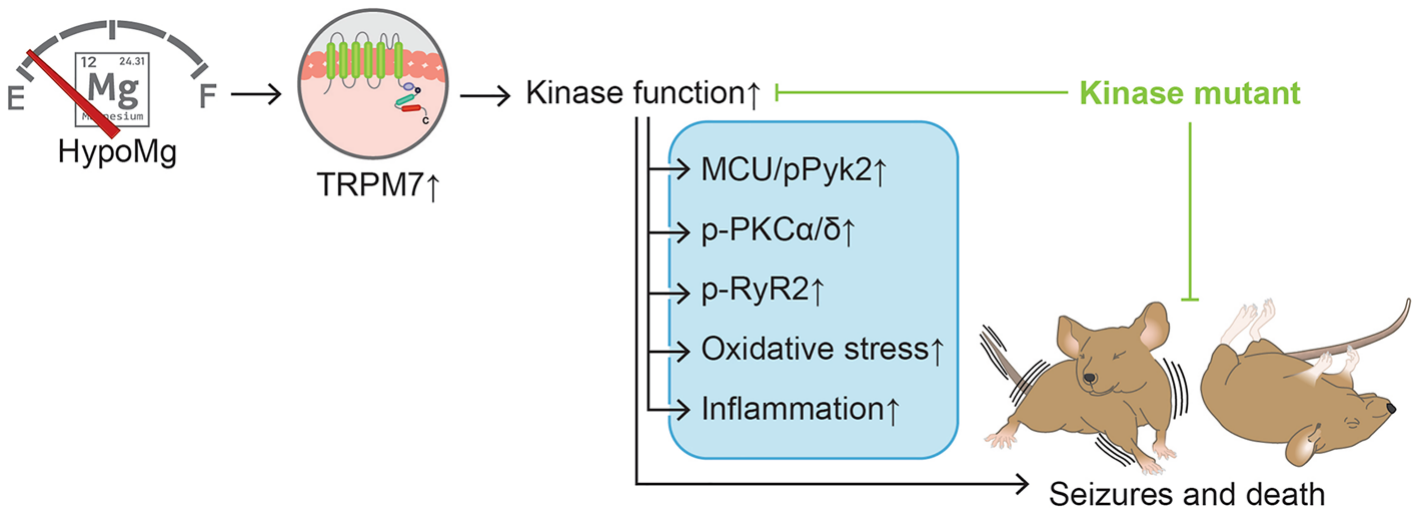
A summarized scheme of how HypoMg causes seizures and death. Upregulated TRPM7 kinase function by HypoMg induces seizures and death in mice, accompanied with kinase phosphorylation (p-PKCα/δ and p-Pyk2), RyR2 phosphorylation (p-RyR2), MCU overexpression, inflammation and oxidative stress in the brain. Inhibition of TRPM7 kinase by a K1646R mutation prevents all these HypoMg-induced changes. *HypoMg* hypomagnesemia, *TRPM7* transient receptor potential cation channel subfamily M 7, *p-PKC* phosphorylated protein kinase C, *p-RyR2* phosphorylated ryanodine receptor 2, *MCU* mitochondrial calcium uniporter, *p-Pyk2* phosphorylated protein tyrosine kinase 2; ↑, increased; Ͱ, inhibition.

TRPM7 kinase is central to a signaling cascade that results in seizures and death in response to HypoMg. A summarized scheme of HypoMg impacts, and how these are altered by inhibiting TRPM7 kinase activity, is shown in Fig. 6. TRPM7 kinase or its downstream mediators may represent novel mediators of seizures and epilepsy associated with HypoMg.

## Methods

Any supporting data not available within the article are available from the corresponding author upon reasonable request.

### Reagents

Chemicals and reagents were purchased from Sigma-Aldrich (St. Louis, MO), except as stated otherwise.

### Study ethics declarations

Animal care and interventions were undertaken in accordance with the National Institute of Health Guide for the Care and Use of Experimental Animals, and the animal protocol (IACUC-2003-37940A) was approved by the Institutional Animal Care and Use Committees of the University of Minnesota. The study was carried out in compliance with the ARRIVE guidelines.

### Animals

WT C57BL/6J mice purchased from the Jackson Laboratory (Bar Harbor, ME) and a C57BL/6J mouse strain with a global homozygous K1646R mutation in the TRPM7 kinase domain, TRPM7^K1646R^ with no kinase function, were fed with a normal Mg diet (control: 2000 mg/kg Mg, Teklad global 18% protein rodent diet 2018, Envigo Teklad Diets, Madison, WI) or low-Mg diet (HypoMg: ~ 15–30 mg/kg Mg, TD.93106, Envigo Teklad Diets) for 4 or 6 weeks. Distilled and deionized water (ddH_2_O) was given to all the mice to control any possible Mg intake from drinking water. This diet was chosen based on previous animal studies^11,53^. TRPM7^K1646R^ was a generous gift from Dr. Bebhinn Treanor (Dept. of Biological Sciences, University of Toronto Scarborough, Toronto, Canada). The lysine 1646 is essential for Mg-ATP binding and its replacement with arginine results in a complete loss of TRPM7 kinase activity while maintaining the channel function of ion transport^24,54^. TRPM7^K1646R^ mice do not show any signs of imbalanced Mg homeostasis at baseline with unaltered Mg concentrations in the blood serum, bones, and platelets^55^. Histological analysis of different organs from TRPM7^K1646R^ mice revealed no obvious alterations, and the mice display no notable anatomical or physiological abnormalities^24,55^. PCR was performed with tail DNA samples to confirm the K1646R mutation for a T->C replacement (CTT for lysine and CCT for arginine) as described^24^. Primers include 5ʹ-AAT GGG AGG TGG TTT ACG-3ʹ and 5ʹ-CTC AGA TCA CAG CTT ACA GTC A-3ʹ. Both TRPM7^K1646R^ and WT allele resulted in a 205 bp amplicon, but WT PCR product could be digested with Msel (aka Tru1l, Thermo Fisher Scientific, Waltham, MA) at 37 °C for 2 h resulting in a 120 and 85 bp fragments.

### Video recording of seizures

Mice were housed in standard housing conditions (12 h light, 12 h dark) in the animal facility at the University of Minnesota and were allowed ad libitum access to food and water. Two cages of mice were video recorded together with a webcam with night vision: one cage of control mice under normal diet and another cage of the same age mice under the low-Mg diet. Animals were checked twice daily, and any mice died were removed from the cages.

### Brain tissue, serum, and urine Mg levels

Brain tissue was weighed (for data normalization) and extracted with Magnesium Assy Buffer (from Magnesium Assay kit, Sigma-Aldrich). Clear extract containing free Mg was obtained by centrifugation at 16,000×*g* for 10 min at 4 °C. Blood was collected in tubes with no additives (BD Microtainer Capillary, Becton, Dickinson and Company, Franklin Lakes, NJ) and was allowed to clot at room temperature for 15–30 min. Serum was obtained by blood centrifugation at 1700×*g* for 10 min at 4 °C. Urine was collected with autoclaved Eppendorf. Brain tissue, serum and urine Mg was measured with the Magnesium Assay kit by following the manufactory’s instruction.

### Western blot

After 4 or 6 weeks of control or low-Mg diet, age- and sex-matched mice were deeply anesthetized with 5% isoflurane. The brain or hippocampus tissue was dissected, snap frozen in liquid nitrogen, and stored at − 80 °C. To make protein lysate, the snap frozen brain or hippocampus was suspended and cut into small pieces in T-PER™ Tissue Protein Extraction reagent (ThermoFisher Scientific 78510) containing protease/phosphatase inhibitors (~ 5 mL/g of tissue). Tissues were then disrupted using blender on ice (5 s for 3 times) and incubated on ice for 10 min. Protein lysates were obtained from the supernatant by centrifugation at 10,000×*g* for 10 min. Total protein concentration of the supernatant was measured with the Pierce BCA Protein Assay Kit (ThermoFisher Scientific 23225). After mixing with 4× NuPAGE LDS Sample Buffer/β-mercaptoethanol (1:10 dilution), the protein lysate was de-natured at 95 °C for 5 min. Proteins were separated on SDS-PAGE gels and transferred onto methanol-activated polyvinylidene difluoride (PVDF) membrane for 2 h at 65 mV and 4 °C. Following 5% nonfat dry milk or 5% bovine serum albumin in Tris-buffered saline, 0.1% Tween 20 blocking for 1 h, the membranes were incubated with the primary antibodies overnight at 4 °C. The following antibodies were used: anti-TRPM7 (Alomone ACC-047), c-Fos (Cell Signaling 2250, Danvers, MA), anti-nitro-tyrosine (Abcam 7048, Cambridge, MA), anti-NLRP3 (Abcam 263899), anti-ASC (Cell Signaling 67824), anti-IL-1β (Novus Bio NBP1-42767, Centennial, CO), anti-p-PKCα (Abcam 76016), anti-p-PKCδ (Cell Signaling 9374), anti-MCU (Cell Signaling 14997), anti-p-Pyk2 Tyr402 (Cell Signaling 3291), anti-p-Pyk2 Tyr579/580 (ThermoFisher Scientific 44-636G), anti-p-RyR2 S2808 and S2814 (Badrilla A01-30AP and A010-31AP, Leeds, UK), anti-CNNM2 (ThermoFisher Scientific PA5-102013), anti-MRS2 (Novus Bio NBP2-34200), anti-SLC41A1 (ThermoFisher Scientific PA5-53286), anti-SLC41A3 (Novus Bio NBP1-59764), and anti-p-Src Ser17 (Cell Signaling 12432). Horseradish peroxidase-conjugated goat anti-rabbit and anti-mouse IgG secondary antibodies (Bio-Rad, Hercules, CA) were be used at 1:4000 dilution for 1 h at room temperature. GAPDH and α-tubulin (Abcam 9484 and 176560) were used as loading controls. Optical density of the bands was measured with ChemiDoc MP system (Bio-Rad) and analyzed with Image Lab 6.0.0 software (Bio-Rad).

### Telemetry

Cardiac rhythm was monitored using telemetry devices. Briefly, mice were implanted with ETA-F10 transmitter (Data Science International, St. Paul, MN) as we have done previously^56^. Under 1% isoflurane anesthesia, a skin incision was made at the dorsal neck area and a transmitter was inserted subcutaneously. The two ECG leads were tunneled under skin and positioned to generate a lead II ECG configuration. Continuous ECG recording was initiated immediately after transmitter implantation using Dataquest ART software (Version 4.1, DSI), and lasted until the occurrence of animal death or the end of 6-week low-Mg diet. The ECG prior to the animal death was analyzed to determine the cause of death.

### Statistics

Data are presented as mean ± standard deviation (SD). For the dot plots, the lines indicated the mean values, and the error bars indicated SEM values. GraphPad Prism 9.6 (GraphPad Software Inc., San Diego, CA) was used for statistical analysis. The Two-way ANOVA for multiple groups’ comparison was used. Figure 1B and Supplementary Fig. S2 used the Kaplan Meier plot for the survival analysis of mice with control and low-Mg diet and the log-rank (Mantel–Cox) test was applied for comparison between the groups. A P value of < 0.05 was considered statistically significant.

## Supplementary Information

Supplementary Information 1.

Supplementary Information 2.

Supplementary Video 1.

## Data Availability

All data are available in the main text, the Supplementary Materials, or from the contact author, Dr. Samuel Dudley at sdudley@umn.edu.
