# Supplementary material for: TRPM7 kinase mediates hypomagnesemia-induced seizure-related death
Source: Sci Rep. 2023 May 15;13:7855. doi: 10.1038/s41598-023-34789-2 (PMC10185534; doi:10.1038/s41598-023-34789-2)
Supplement: Supplementary file 1 — Supplementary Information 1. [file 41598_2023_34789_MOESM1_ESM.pdf]

Supplementary Materials:

Figure S1. A representative trace of telemetry ECG shows possible seizure prior to death. A similar pattern was observed in all ECG recorded deaths (n=6 of 14 mice).

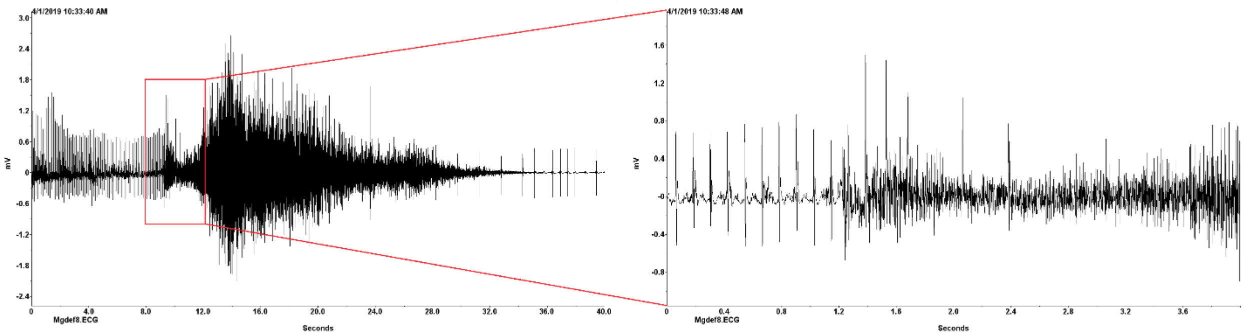

Figure S2. The Kaplan Meier survival curves of the WT mice on normal (WT-control) and low Mg diet (WT-HypoMg male and WT-HypoMg female). The Log-rank (Mantel-Cox) test gave a P=0.0007 when comparing WT-control and WT-HypoMg male, P<0.0001 when comparing WT-control and WT-HypoMg female, and P<0.0001 when comparing WT-HypoMg male and WT-HypoMg female. The number of mice are 25 for WT-control on the normal diet (12 males and 13 females), 40 for WT-HypoMg males and 48 for WT-HypoMg females on the low-Mg diet. Data are from our previous work[10].

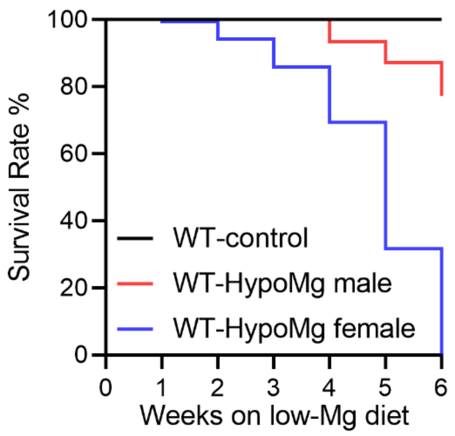

Supplementary Video-1. Representative video clips confirm seizure activity immediately prior to death, indicating prolonged status epilepticus and sudden death of WT mice under the low Mg diet (15-30 mg/kg Mg, ddH_2O, for up to 6 weeks, starting at 10 weeks old, the cage on the right side). We recorded seizure and death of two male mice and one female mouse out of 7 mice (3 males and 4 female). There were variable amounts of time between the start of tonus and death, with some as short as 15 s (as in the third video clip) and with some having a period of status epilepticus as long as 12 min (as in the first video clip, video showed 1:30 min, longer video is available under request). Control mice with normal diet (2 g/kg Mg, the cage on the left side) were recorded at the same time for comparison.
