# Supplementary material for: TRPM7 kinase mediates hypomagnesemia-induced seizure-related death
Source: Sci Rep. 2023 May 15;13:7855. doi: 10.1038/s41598-023-34789-2 (PMC10185534; doi:10.1038/s41598-023-34789-2)
Supplement: Supplementary file 2 — Supplementary Information 2. [file 41598_2023_34789_MOESM2_ESM.pdf]

Full, uncropped gel and blot images with ladder markers:

Figure 1(d)

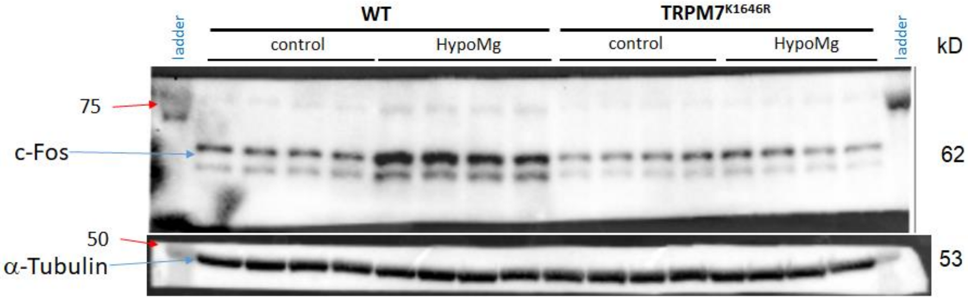

Figure 2

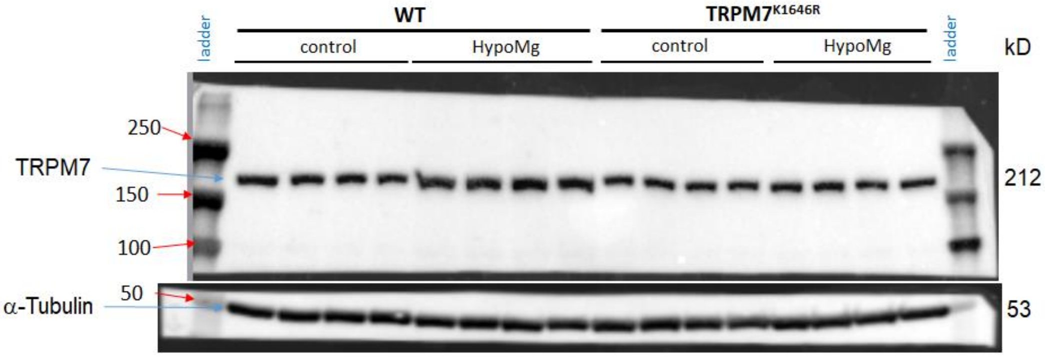

Figure 3(a)

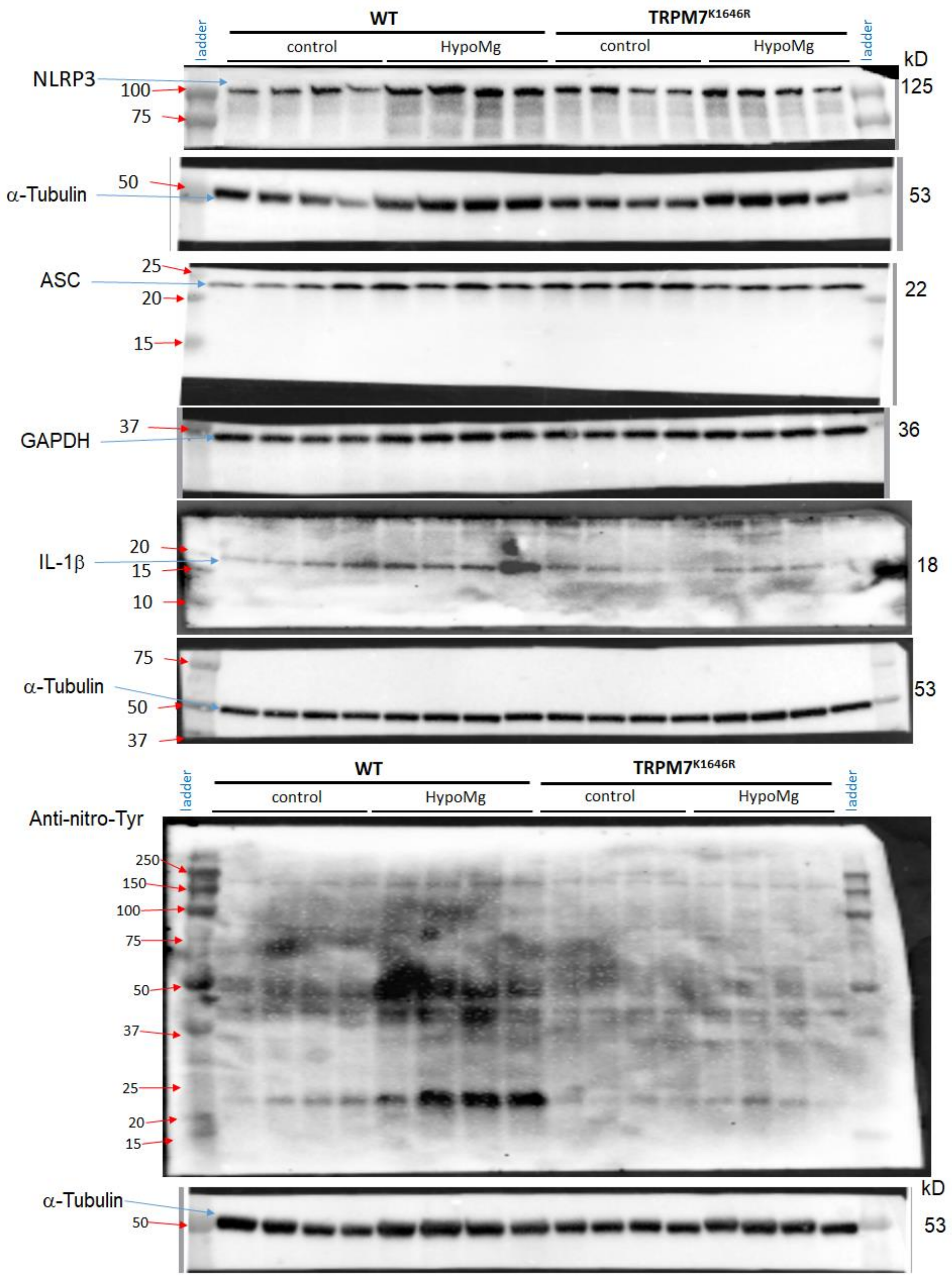

WT
TRPM7K1646R
control
HypoMg
control
HypoMg
ladder
ladder
kD
NLRP3
100
75
125
α-Tubulin
50
53
ASC
25
20
15
22
GAPDH
37
36
IL-1β
20
15
10
18
α-Tubulin
75
50
37
53
WT
TRPM7K1646R
control
HypoMg
control
HypoMg
ladder
ladder
Anti-nitro-Tyr
250
150
100
75
50
37
25
20
15
α-Tubulin
50
kD
53

Figure 4(a)

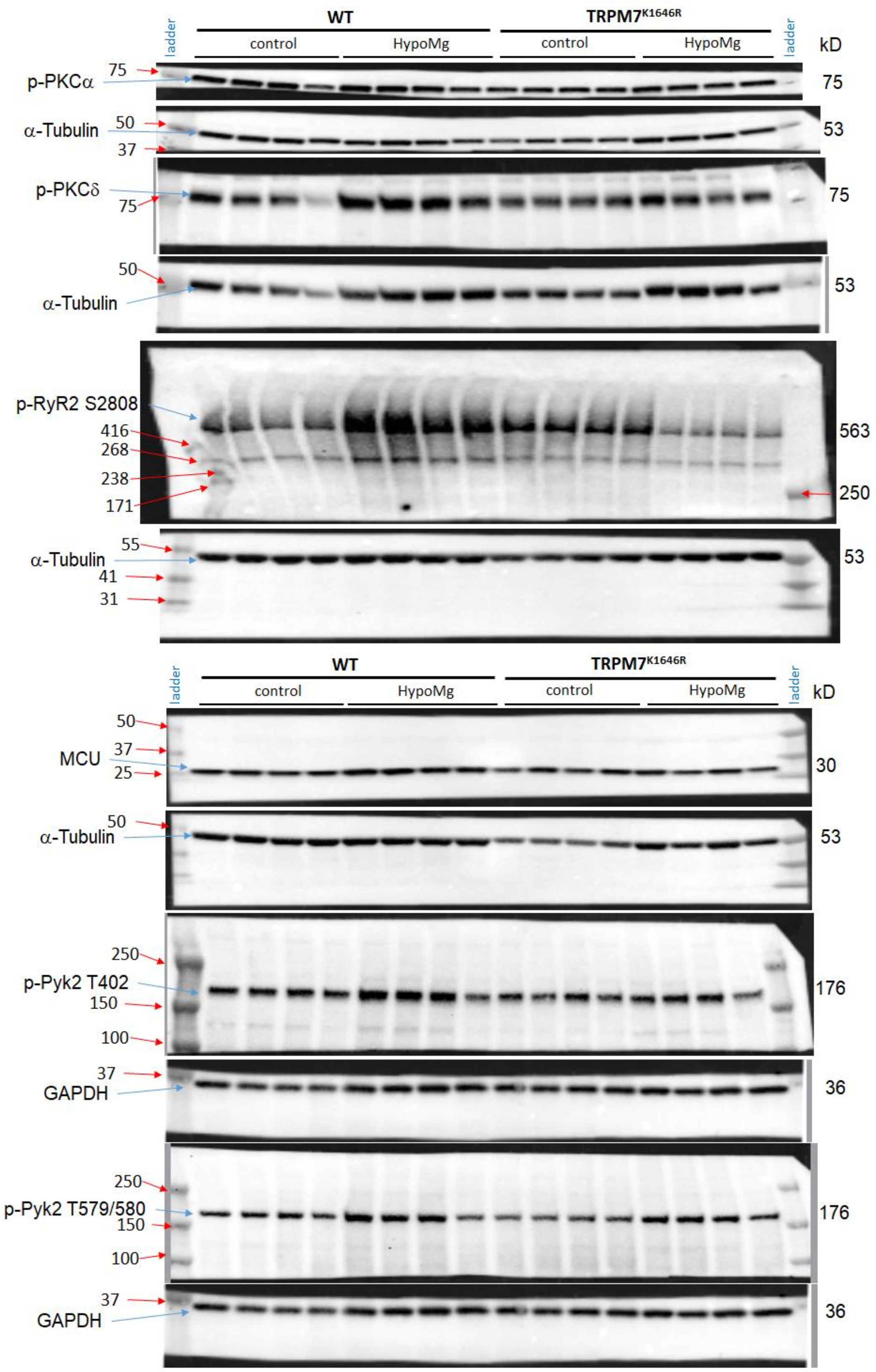
WT
TRPM7K1646R
control
HypoMg
control
HypoMg
ladder
ladder
kD
p-PKCα
75
75
α-Tubulin
50
37
53
p-PKCδ
75
75
α-Tubulin
50
53
p-RyR2 S2808
416
268
238
171
563
250
α-Tubulin
55
41
31
53
WT
TRPM7K1646R
control
HypoMg
control
HypoMg
ladder
ladder
kD
MCU
50
37
25
30
α-Tubulin
50
53
p-Pyk2 T402
250
150
100
176
GAPDH
37
36
p-Pyk2 T579/580
250
150
100
176
GAPDH
37
36

Figure 5 a and b

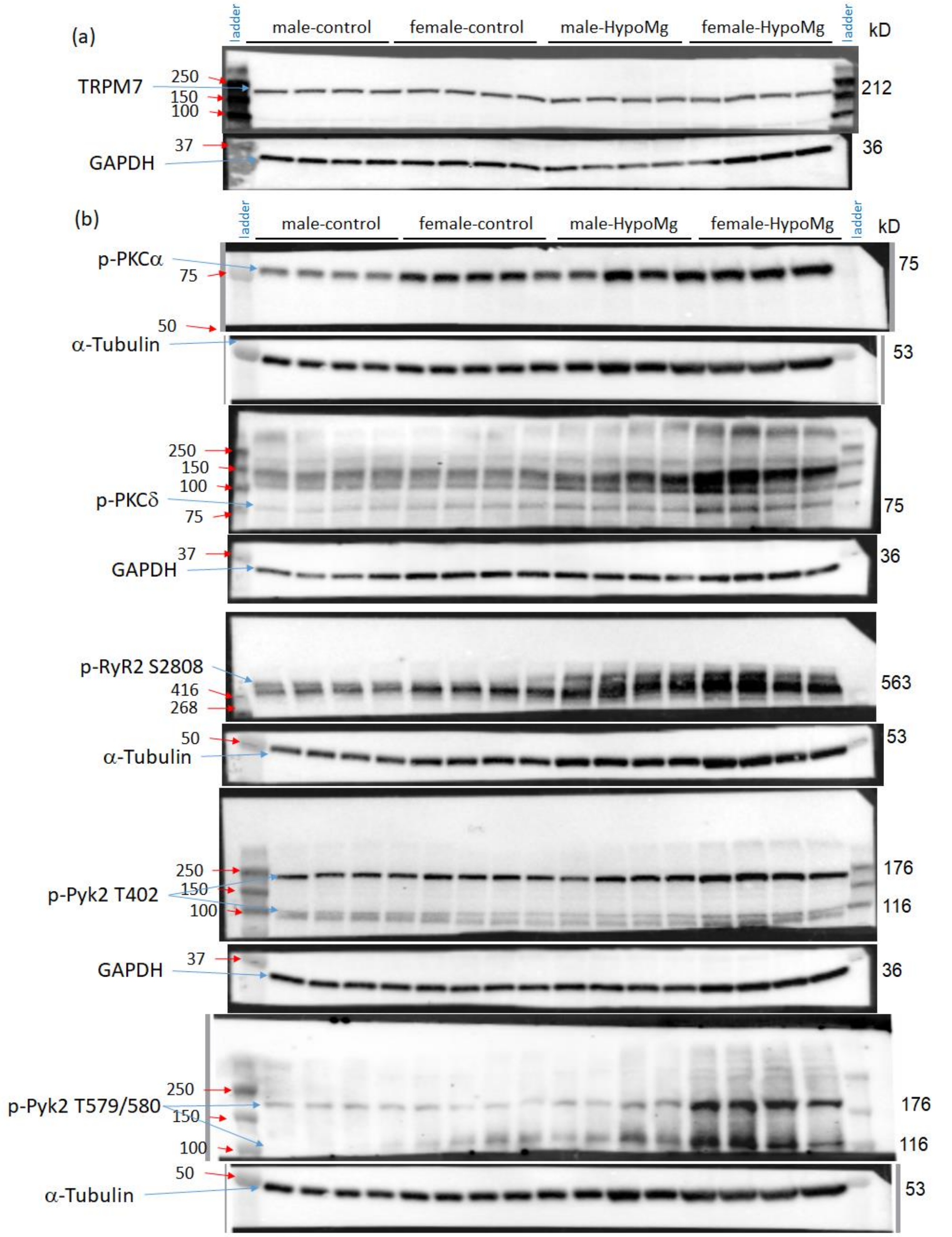

Fig 5 h and i

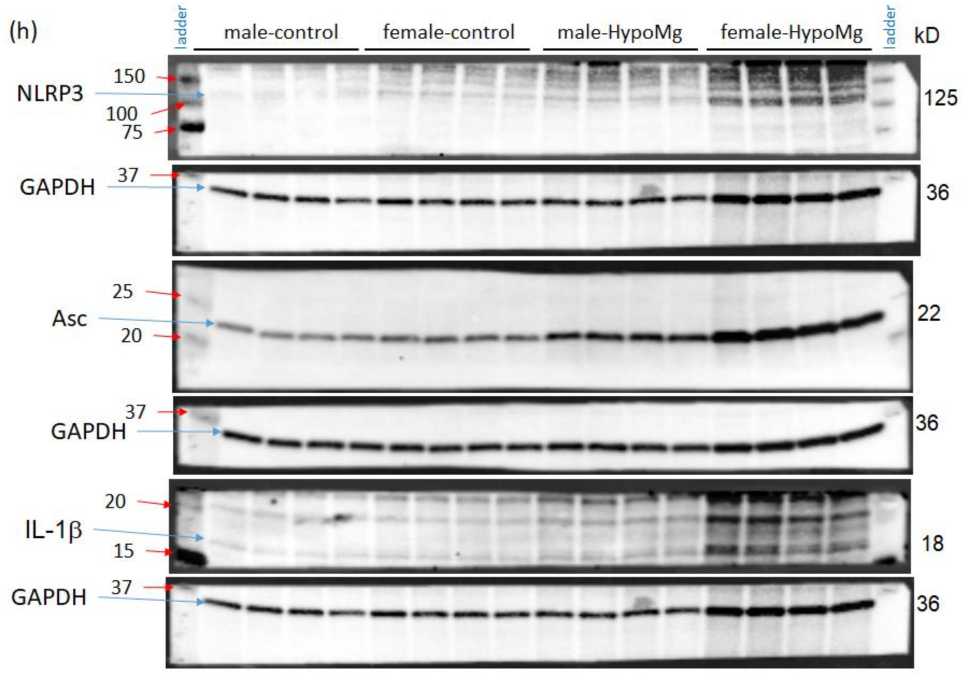

(h)
ladder
male-control
female-control
male-HypoMg
female-HypoMg
ladder
kD
NLRP3
150
100
75
125
GAPDH
37
36
Asc
25
20
22
GAPDH
37
36
IL-1β
20
15
18
GAPDH
37
36

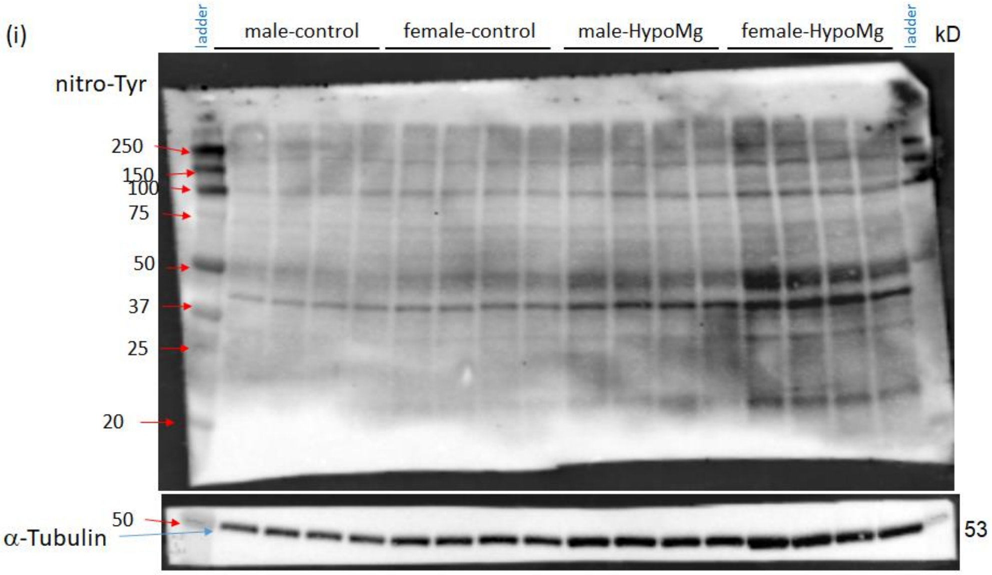

(i)
ladder
male-control
female-control
male-HypoMg
female-HypoMg
ladder
kD
nitro-Tyr
250
150
100
75
50
37
25
20
α-Tubulin
50
53
